# First‐line cetuximab + platinum‐based therapy for recurrent/metastatic head and neck squamous cell carcinoma: A real‐world observational study—ENCORE

**DOI:** 10.1002/cnr2.1804

**Published:** 2023-04-17

**Authors:** Christophe Le Tourneau, Massimo Ghiani, Maria Chiara Cau, Roberta Depenni, Graziana Ronzino, Pierluigi Bonomo, Vincenzo Montesarchio, Luigi Leo, Jeltje Schulten, Satu Salmio, Diethelm Messinger, Andrea Sbrana, Edith Borcoman, Maria Grazia Ghi

**Affiliations:** ^1^ Department of Drug Development and Innovation (D3i) Institut Curie Paris France; ^2^ INSERM U900 Research Unit Saint‐Cloud France; ^3^ Faculty of Medicine Paris‐Saclay University Paris France; ^4^ Department of Oncology Azienda Ospedaliera Brotzu Cagliari Italy; ^5^ Department of Oncology and Hematology Università degli Studi di Modena e Reggio Emilia Modena Italy; ^6^ Oncology Unit Ospedale, Vito Fazzi Lecce Italy; ^7^ Radiation Oncology Azienda Ospedaliero‐Universitaria Careggi Florence Italy; ^8^ AORN dei Colli Monaldi Hospital Naples Italy; ^9^ Global Medical Unit Oncology Merck Healthcare KGaA Darmstadt Germany; ^10^ Statistics Prometris GmbH Mannheim Germany; ^11^ Service of Pneumo‐Oncology Azienda Ospedaliero‐Universitaria Pisana Pisa Italy; ^12^ Department of Surgical, Medical and Molecular Pathology and Critical Care Medicine University of Pisa Pisa Italy; ^13^ Oncology Unit 2 Istituto Oncologico Veneto–IRCCS Padova Italy

**Keywords:** cetuximab, combination chemotherapy, first‐line, observational study, squamous cell carcinoma of head and neck

## Abstract

**Background:**

ENCORE, an observational, prospective, open‐label study, investigated real‐world treatment practices and outcomes with cetuximab plus platinum‐based therapy (PBT) in first‐line (1L) recurrent and/or metastatic squamous cell carcinoma of the head and neck (R/M SCCHN).

**Aims:**

This multinational study aimed to investigate the long‐term use of cetuximab plus PBT for 1L R/M SCCHN in a clinical setting. In particular, this study aimed to explore clinical considerations such as the decision to prescribe cetuximab plus PBT in R/M SCCHN, the mode and duration of treatment, and patient outcomes.

**Methods and Results:**

Previously untreated patients with R/M SCCHN whose planned treatment was cetuximab plus PBT were enrolled from 6 countries. Among 221 evaluable patients, planned treatments included cetuximab plus carboplatin (31.2%), cisplatin plus 5‐fluorouracil (31.7%), or carboplatin plus 5‐fluorouracil (23.1%); 3.2% included a taxane, and 45.2% did not include 5‐fluorouracil. Cetuximab treatment was planned for a fixed duration (≤24 weeks) in 15 patients (6.8%) and until disease progression in 206 (93.2%). Median progression‐free survival and overall survival were 6.5 and 10.8 months, respectively. Grade ≥3 adverse events occurred in 39.8% of patients. Serious adverse events occurred in 25.8% of patients; 5.4% were cetuximab‐related.

**Conclusion:**

In patients with R/M SCCHN, first‐line cetuximab plus PBT was feasible and modifiable in a real‐world setting with similar toxicity and efficacy as in the pivotal phase III EXTREME trial. Clinical trial registration number: EMR 062202‐566.

## 1 INTRODUCTION

Recurrent and/or metastatic (R/M) disease develops in approximately 45% to 65% of patients with squamous cell carcinoma head and neck cancer (SCCHN); these patients have a very poor prognosis and a median overall survival (OS) of <1 year.^1^, ^2^ Factors such as treatment resistance, consequent recurrence of the tumor, and lymph node metastases all contribute to poor OS rates.^3^ Additionally, the epidermal growth factor receptor (EGFR) is overexpressed in up to 90% of patients with SCCHN and is associated with decreased survival.^4^ Cetuximab, the only immunoglobulin G1 monoclonal antibody approved for the treatment of SCCHN,^4^ binds to the extracellular domain of the EGFR, thus preventing receptor activation and subsequent downstream signaling, leading to tumor cell apoptosis.^4^, ^5^ Furthermore, cetuximab can stimulate antibody‐dependent cellular cytotoxicity, which leads to the priming and activation of immune effector cells and facilitates immune cell infiltration into the tumor microenvironment.^5^

The randomized phase III EXTREME trial established the EXTREME regimen (cetuximab plus platinum plus 5‐fluorouracil [5‐FU] for up to 6 cycles, followed by cetuximab maintenance until progressive disease [PD]), as the first regimen in approximately 30 years to significantly improve disease control and survival versus chemotherapy for first‐line (1L) R/M SCCHN.^6^ The addition of cetuximab to a standard chemotherapy regimen led to an increased overall response rate (ORR) from 20% to 36%, improved median progression‐free survival (PFS) from 3.3 to 5.6 months, and improved median OS from 7.4 to 10.1 months versus chemotherapy alone.^6^ Since its approval for the treatment of SCCHN over a decade ago, cetuximab has been widely used in clinical practice in the real‐world setting; the NCCN Clinical Practice Guidelines in Oncology (NCCN Guidelines®) recommend cetuximab plus platinum plus 5‐FU as one of the 1L treatment options for R/M SCCHN with category 1 evidence.^7^ Recently, alternative agents and regimens have become available for the treatment of 1L R/M SCCHN. Results from the large, randomized TPExtreme study support the use of the platinum‐based TPEx regimen, which substitutes 5‐FU in the EXTREME regimen for a taxane (docetaxel), as an alternative 1L treatment option for patients with R/M SCCHN.^8^ Advantages of the TPEx regimen include convenience due to fewer cycles of chemotherapy, less toxicity, and encouraging survival results compared with the EXTREME regimen.^8^ However, questions still remain about its hematological toxicity profile and the best sequential treatment.^9^ Pembrolizumab, a programmed cell death receptor 1 (PD‐1)‐blocking antibody, was approved by the US Food and Drug Administration (FDA) for the 1L treatment of R/M SCCHN in 2019 based on the results of the KEYNOTE‐048 trial.^10^, ^11^ The European Commission approval of pembrolizumab for 1L R/M SCCHN indicates its use as a monotherapy or in combination with platinum and 5‐FU in patients whose tumors express programmed cell death‐ligand 1 (PD‐L1) with a combined positive score of ≥1.^12^ The NCCN guidelines® recommend the EXTREME regimen as one of the 1L treatment options for patients with R/M SCCHN, regardless of PD‐L1 status.^7^ Cetuximab combined with pembrolizumab is also being explored for R/M SCHNN and clinical trials have shown promising preliminary results; a study in 33 patients who had not received any previous PD‐1, PD‐L1, or EGFR inhibition demonstrated an ORR of 45% for this combination.^13^

Although recent clinical trials have provided insight into the use of cetuximab plus platinum‐based therapy (PBT) in a controlled setting, little is known about its use in the real‐world clinical setting by clinicians in Europe, particularly for those patients who are typically excluded from randomized controlled trials (e.g., patients who have an Eastern Cooperative Oncology Group performance status [ECOG PS] score of >2 or are elderly). Observational studies are therefore necessary to ensure that benefits observed in controlled clinical trials translate accordingly to patients in the real‐world setting. ENCORE (prospEctive observatioNal Clinical practice study in the 1st line management Of Recurrent/metastatic SCCHN with Erbitux and chemotherapy; EMR 062202‐566) was a multinational (Algeria, France, Italy, Portugal, Russia, and South Africa), observational, prospective, open‐label study that investigated the real‐world treatment practices, efficacy, and safety of cetuximab plus PBT in 1L R/M SCCHN. The ENCORE study aimed to investigate the widespread use of cetuximab plus PBT for R/M SCCHN in a clinical setting over many years.

In particular, the ENCORE study aimed to explore clinical considerations regarding cetuximab plus PBT usage in R/M SCCHN, such as the decision to prescribe, the mode and duration of treatment, and patient outcomes. This study aimed to create one centralized database with the intention of evaluating results from different regions and countries, thereby permitting a descriptive comparison across the selected countries.

## 2 METHODS

### 2.1 Study design

ENCORE was a post authorization, non‐interventional, prospective, non‐comparative and open‐label phase IV study that investigated the use of cetuximab plus PBT in 1L R/M SCCHN. Patients were eligible if they were ≥18 years of age; had confirmed SCCHN of the oral cavity, oropharynx, hypopharynx, or larynx with recurrence not amenable to curative locoregional therapy and/or distant metastases; were suitable for 1L combination treatment with cetuximab plus PBT with or without local palliative treatments; and had a planned treatment regimen that included cetuximab. Patients were excluded from the study if they had previously received chemotherapy for R/M SCCHN (however, previous adjuvant or neoadjuvant chemotherapy was acceptable), or if they were not considered suitable for the treatment and the study, as per normal clinical practice for 1L treatment of R/M SCCHN at the investigator's site in the participating countries. The study planned to enroll up to 300 patients, or the maximum number of eligible patients in the participating countries by December 2016.

Treatment involved cetuximab in combination with PBT for the 1L treatment of R/M SCCHN. All agents were given according to their locally approved labels, and planned treatment duration was according to normal clinical practice for each treating physician. The clinical decision to use cetuximab with PBT and the recording of patient baseline characteristics, planned treatment regimens, administration schedule, and efficacy/safety outcomes were at the sole discretion of the investigator. Patient follow‐up was for 1 year after the first dose of cetuximab, or until death, whichever occurred first (some patients had longer follow‐ups as the follow‐up period was initially planned at 2 years and was reduced to 1 year after a protocol amendment; additionally, some patients began cetuximab treatment prior to enrollment). Patients were considered as receiving cetuximab maintenance therapy if any administration of cetuximab was given >7 days after the last administration of chemotherapy.

The primary objective was to assess treatment patterns for cetuximab, especially duration of use, for the treatment of 1L R/M SCCHN in combination with platinum‐based chemotherapy across different regions worldwide. Key secondary objectives were to assess treatment outcomes, including the endpoints of best overall response (BOR), disease control rate (DCR), ORR, PFS and OS, and adverse events (AEs) related to cetuximab. Only serious adverse events (SAEs), non‐serious AEs related to study treatment, and AEs leading to a delay or the permanent discontinuation of study treatment were to be recorded until the 30‐day posttreatment safety follow‐up assessment; AEs were graded according to the National Cancer Institute Common Terminology for Adverse Events v4.03.

Because this was a prospective observational study, several patients were enrolled who had started cetuximab treatment prior to enrollment; these patients were included in the analysis providing that consent had been given and that retrospective data were available. The full analysis set (FAS) therefore included all enrolled patients who received cetuximab; primary endpoints and safety were analyzed in the FAS. As some patients began cetuximab treatment prior to enrollment, the target analysis set (TAS) was created to allow for output generation of patients in the FAS. The TAS included all patients who received their first dose of cetuximab on or after the date of enrollment. Secondary efficacy endpoints were analyzed in both the FAS and TAS.

### 2.2 Statistical analyses

Descriptive and exploratory statistical methods were used to analyze the study data; all statistical methods were of exploratory nature only.

BOR, ORR and DCR: these qualitative variables were summarized as a number and percentage for the FAS and TAS. The Cochran–Mantel–Haenszel test was used to test for differences in ORR and DCR between patients with planned cetuximab treatment until PD versus those with a planned fixed duration of cetuximab treatment. The corresponding odds ratios and 95% confidence intervals (CIs) for the odds ratios were determined. The association between ORR and planned duration of cetuximab treatment, respectively, and possible explanatory variables were analyzed using logistic regression methods.

PFS and OS: The Kaplan–Meier method was used to estimate median PFS and OS and the corresponding 95% CIs. A log‐rank test was used to compare PFS and OS between patients who received cetuximab until PD and those who received cetuximab for a fixed duration. The hazard ratios for PFS and OS (including the 95% CIs) were determined to assess the effect of planned cetuximab treatment duration (not until PD vs. until PD). Cox proportional hazard methods were used to investigate the association between PFS and OS and possible explanatory variables.

Due to a protocol amendment, the planned follow‐up time was reduced from 2 years to 1 year to allow for an earlier study end. However, the follow‐up date for patients enrolled under the original plan of 2 years of follow‐up was not censored; all evaluable data were used.

## 3 RESULTS

### 3.1 Planned treatments and patient baseline characteristics

**FIGURE 1:**
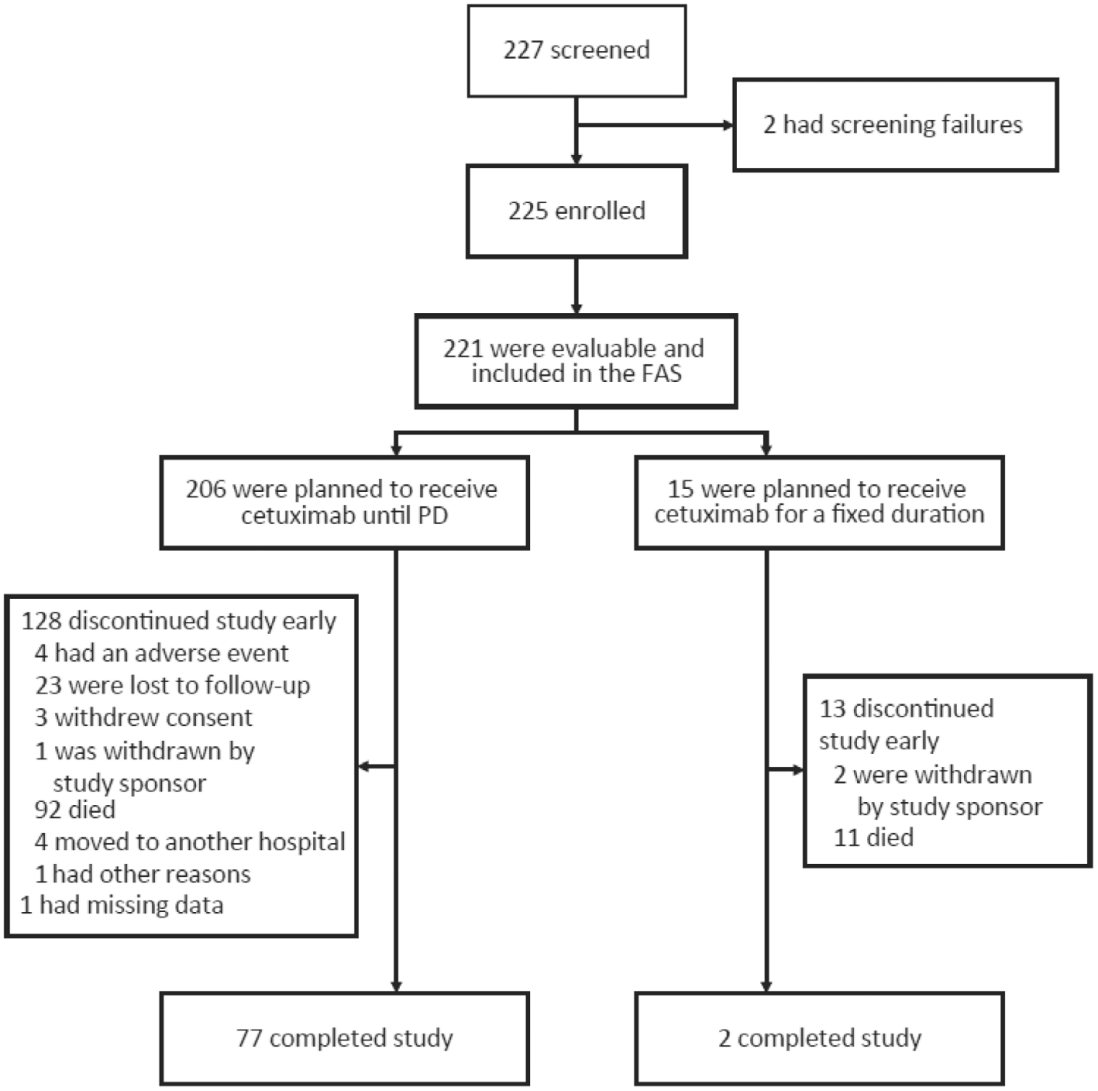
Study profile. FAS, full analysis set; PD, progressive disease.

A total of 225 patients were enrolled; 221 patients received ≥1 dose of cetuximab and were included in the FAS and the safety population (Figure 1), while the remaining four patients did not receive cetuximab and were excluded. Patients were from Algeria (*n* = 1 [0.5%]), France (*n* = 46 [20.8%]), Italy (*n* = 144 [65.2%]), Portugal (*n* = 8 [3.6%]), Russia (*n* = 21 [9.5%]), and South Africa (*n* = 1 [0.5%]). The enrollment period was approximately 1.5 years, with the first patient enrolled in May 2015 and the last patient enrolled in December 2016. A total of 159 patients in the FAS (71.9%) with prospective data collection were included in the TAS. Most patients had a follow‐up of 1 year; however, patients who began cetuximab treatment before enrollment, or were enrolled >1 year before the protocol amendment, had longer follow‐ups. Baseline characteristics are presented in Table 1. Tumors were not evaluated for human papilloma virus (HPV) status in 86.4% of patients in the FAS; of the total 56 patients in the FAS with oropharyngeal cancer, 16 (28.6%) had an HPV test performed.

**TABLE 1:** Baseline characteristics in the FAS.

Characteristic	Cetuximab until PD (*n* = 206)	Cetuximab fixed duration (*n* = 15)	Total (*N* = 221)
Male, *n* (%)	158 (76.7)	10 (66.7)	168 (76.0)
Age, median (range), years	64 (22–91)	61 (31–80)	64 (22–91)
*ECOG PS score, n (%)*			
0	66 (32.0)	4 (26.7)	70 (31.7)
1	109 (52.9)	9 (60.0)	118 (53.4)
2	27 (13.1)	2 (13.3)	29 (13.1)
3	1 (0.5)	0	1 (0.5)
Missing	3 (1.5)	0	3 (1.4)
*Previous treatment, n (%)*			
Cetuximab	24 (11.7)	7 (46.7)	31 (14.0)
Radiotherapy	153 (74.3)	15 (100.0)	168 (76.0)
Chemotherapy	109 (52.9)	8 (53.3)	117 (52.9)
Surgery	113 (54.9)	7 (46.7)	120 (54.3)
None	25 (12.1)	0	25 (11.3)
*Disease status, n (%)*			
Locoregional recurrence; no metastases	97 (47.1)	11 (73.3)	108 (48.9)
Locoregional recurrence; metastases	37 (18.0)	3 (20.0)	40 (18.1)
No locoregional recurrence; metastases	72 (35.0)	1 (6.7)	73 (33.0)
Time since diagnosis of R/M SCCHN, median (range), months	1.65 (0–100.8)	2.0 (0.1–16.4)	1.70 (0–100.8)
*Primary tumor site, n (%)* ^a^			
Hypopharyngeal	33 (16.0)	4 (26.7)	37 (16.7)
Laryngeal	53 (25.7)	0	53 (24.0)
Oral cavity	62 (30.1)	1 (6.7)	63 (28.5)
Oropharyngeal	46 (22.3)	10 (66.7)	56 (25.3)
Other	18 (8.7)	2 (13.3)	20 (9.0)

Abbreviations: ECOG PS, Eastern Cooperative Oncology Group performance status; FAS, full analysis set; PD, progressive disease; R/M, recurrent/metastatic; SCCHN, squamous cell carcinoma of the head and neck.

^a^ Based on prespecified terms; recording of multiple locations for the primary tumor site was possible.

The most commonly planned treatments for the ENCORE patient population included chemotherapy backbones consisting of cisplatin plus 5‐FU (*n* = 70 [31.7%]), carboplatin plus 5‐FU (*n* = 51 [23.1%]), or carboplatin alone (*n* = 69 [31.2%]). A taxane was included in 3.2% of planned treatments. In total, 45.4% of planned treatments did not include 5‐FU. In the FAS, cetuximab treatment was planned until PD for most patients (*n* = 206 [93.2%]), of which chemotherapy was planned until PD for 111 (53.9%), and for a fixed duration (3–30 weeks) for 95 (46.1%); both cetuximab and chemotherapy were planned for a fixed duration of ≤24 weeks for 15 patients (6.8%) (Supplementary Table 1). The treatment goal was palliative, potentially curative, and symptomatic treatment in 141 patients (63.8%), 65 (29.4%), and 15 (6.8%), respectively, in the FAS.

### 3.2 Treatments received

Cetuximab plus PBT regimens received in the overall population (FAS) are outlined in Table 2. A total of 90 patients (40.7%) received cisplatin either alone or in combination with 5‐FU, capecitabine, or docetaxel; 130 patients (58.8%) received carboplatin either alone or in combination with 5‐FU, paclitaxel, capecitabine, docetaxel, or gemcitabine; 1 patient (0.5%) received paclitaxel alone; and the chemotherapy regimen was not specified for 2 patients (0.9%). Within the study, the median duration of chemotherapy and cetuximab was 14 weeks (range, 1–92 weeks) and 19 weeks (range, 1–263 weeks), respectively. A total of 132 patients (59.7%) received cetuximab maintenance; median duration was 8 weeks (range, 1–98 weeks). In the FAS, the cetuximab dose was reduced in 21 patients (9.5%) and permanently discontinued in 174 (78.7%). The most common reasons for cetuximab discontinuation were disease progression (*n* = 72 [32.6%]), occurrence of SAE (*n* = 12 [5.4%]), investigator decision (*n* = 25 [11.3%]), treated according to a planned fixed duration (*n* = 13 [5.9%]), and death (*n* = 11 [5.0%]) (Supplementary Table 2). The mean total number of cetuximab administrations was 14.1 ± 12.91 with a cumulative cetuximab dose of 4032.9 ± 3653.64 mg/m^2^, and the mean relative dose intensity was 72.0 ± 30.62% (Supplementary Table 3).

**TABLE 2:** Chemotherapy regimens received in the FAS.

Chemotherapy regimen	Patients, *n* (%)		
	Cetuximab until PD (*n* = 206)	Cetuximab fixed duration (*n* = 15)	Total (*N* = 221)
Cisplatin + 5‐FU	65 (31.6)	5 (33.3)	70 (31.7)
Carboplatin	67 (32.5)	2 (13.3)	69 (31.2)
Carboplatin + 5‐FU	46 (22.3)	5 (33.3)	51 (23.1)
Cisplatin	14 (6.8)	1 (6.7)	15 (6.8)
Cisplatin + capecitabine	4 (1.9)	0	4 (1.8)
Carboplatin + paclitaxel	4 (1.9)	0	4 (1.8)
Carboplatin + capecitabine	3 (1.5)	0	3 (1.4)
Carboplatin + gemcitabine	2 (1.0)	0	2 (0.9)
Cisplatin + docetaxel	0	1 (6.7)	1 (0.5)
Carboplatin + docetaxel	0	1 (6.7)	1 (0.5)
Paclitaxel	1 (0.5)	0	1 (0.5)
Missing	2 (1.0)	0	2 (0.9)

Abbreviations: 5‐FU, 5‐fluorouracil; FAS, full analysis set; PD, progressive disease.

### 3.3 Efficacy outcomes

Efficacy outcomes for the FAS and TAS are presented in Table 3. Among patients in the FAS who received cetuximab until PD (*n* = 206) or for a fixed duration (*n* = 15), a BOR of complete response was achieved by 12 patients (5.8%) and 0 patients, and a BOR of partial response was achieved by 43 patients (20.9%) and 6 patients (40.0%), respectively. Among patients in the TAS who received cetuximab until PD (*n* = 144) or for a fixed duration (*n* = 15), a BOR of complete response was achieved by 6 patients (4.2%) and 0 patients, and a BOR of partial response was achieved by 28 patients (19.4%) and 6 patients (40.0%), respectively. ORR and disease control rate were 27.6% (95% CI, 21.8%–34.0%) and 65.6% (95% CI, 58.9%–71.9%), respectively, in the FAS and 25.2% (95% CI, 18.6%–32.6%) and 57.9% (95% CI, 49.8%–65.6%), respectively, in the TAS. There were 26 (11.8%) patients who discontinued PBT <6 months and may be considered platinum‐refractory and 195 (88.2%) patients who received no platinum or discontinued ≥6 months. The ORR (complete or partial response) was comparable between both subgroups: 23.1% in patients who had discontinued platinum‐based chemotherapy <6 months versus 28.2% in those who had not received or had discontinued platinum‐based chemotherapy ≥6 months (odds ratio 1.31 [95% CI 0.50, 3.43]).

**TABLE 3:** Efficacy outcomes in the FAS and TAS.

Outcome	FAS			TAS		
	Cetuximab until PD (*n* = 206)	Cetuximab fixed duration (*n* = 15)	Total *N* = 221	Cetuximab until PD (*n* = 144)	Cetuximab fixed duration (*n* = 15)	Total *N* = 159
*BOR, n (%)*						
CR	12 (5.8)	0	12 (5.4)	6 (4.2)	0	6 (3.8)
PR	43 (20.9)	6 (40.0)	49 (22.2)	28 (19.4)	6 (40.0)	34 (21.4)
SD	79 (38.3)	5 (33.3)	84 (38.0)	47 (32.6)	5 (33.3)	52 (32.7)
PD	45 (21.8)	4 (26.7)	49 (22.2)	38 (26.4)	4 (26.7)	42 (26.4)
NE	25 (12.1)	0	25 (11.3)	24 (16.7)	0	24 (15.1)
Missing	2 (1.0)	0	2 (0.9)	1 (0.7)	0	1 (0.6)
ORR (95% CI), %^a^	26.7 (20.8–33.3)	40.0 (16.3–67.7)	27.6 (21.8–34.0)	23.6 (16.9–31.4)	40.0 (16.3–67.7)	25.2 (18.6–32.6)
DCR (95% CI), %^b^	65.0 (58.1–71.5)	73.3 (44.9–92.2)	65.6 (58.9–71.9)	56.3 (47.7–64.5)	73.3 (44.9–92.2)	57.9 (49.8–65.6)
Median PFS (95% CI), months	6.8 (5.5–7.9)	5.0 (3.2–8.0)	6.5 (5.4–7.7)	5.4 (4.1–7.2)	5.0 (3.2–8.0)	5.2 (4.3–7.2)
*PFS rate (95% CI), %*						
6‐month	52.2 (45.0–58.9)	40.0 (16.5–62.8)	51.4 (44.5–57.9)	46.2 (37.7–54.3)	40.0 (16.5–62.8)	45.6 (37.5–53.3)
12‐month	23.0 (17.2–29.4)	13.3 (2.2–34.6)	22.3 (16.7–28.4)	18.7 (12.3–26.2)	13.3 (2.2–34.6)	18.1 (12.2–25.1)
24‐month	7.5 (2.5–16.2)	NE	7.0 (2.4–15.1)	4.4 (0.5–15.8)	NE	3.9 (0.5–14.0)
Median OS (95% CI), months	11.2 (9.1–14.3)	9.2 (6.7–13.4)	10.8 (9.1–13.4)	9.4 (8.2–12.4)	9.2 (6.7–13.4)	9.4 (8.2–11.8)
*OS rate (95% CI), %*						
6‐month	72.1 (65.2–77.9)	73.3 (43.6–89.1)	72.2 (65.5–77.8)	67.1 (58.3–74.4)	73.3 (43.6–89.1)	67.6 (59.4–74.5)
12‐month	48.3 (40.4–55.7)	31.1 (10.2–55.0)	46.7 (39.1–53.8)	42.7 (33.2–51.7)	31.1 (10.2–55.0)	40.9 (32.1–49.5)
24‐month	18.4 (8.4–31.4)	NE	17.3 (7.9–29.7)	15.0 (4.8–30.7)	NE	13.6 (4.3–28.1)

Abbreviations: BOR, best overall response; CR, complete response; DCR, disease control rate; FAS, full analysis set; NE, not evaluable; ORR, overall response rate; OS, overall survival; PD, progressive disease; PFS, progression‐free survival; PR, partial response; SD stable disease; TAS, target analysis set.

^a^ ORR = CR + PR.

^b^ DCR = CR + PR + SD.

**FIGURE 2:**
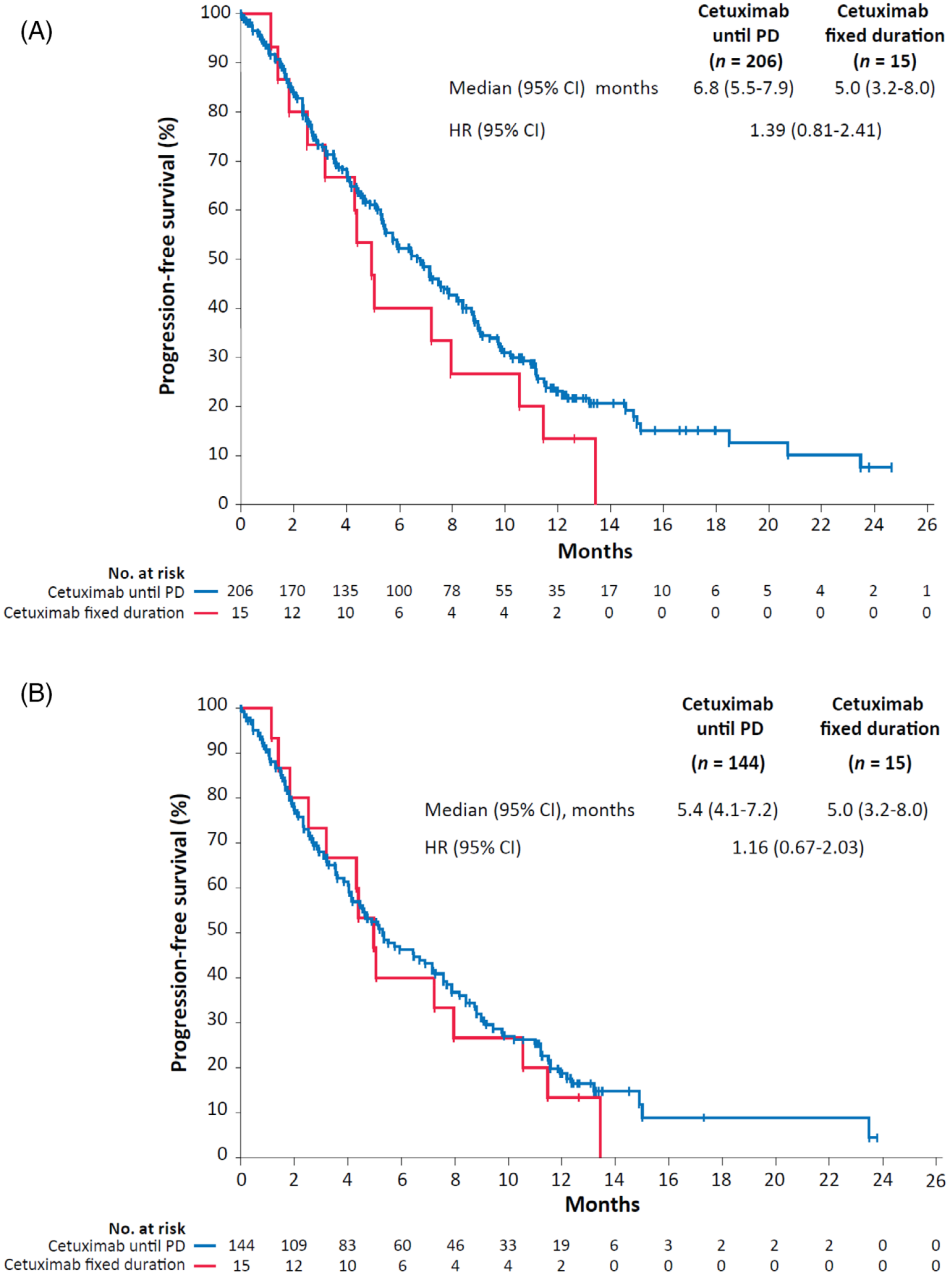
Kaplan–Meier estimates of progression‐free survival for patients whose planned cetuximab treatment was until progressive disease (PD) or for a fixed duration in the full analysis set (A) and target analysis set (B). CI, confidence interval; HR, hazard ratio.

**FIGURE 3:**
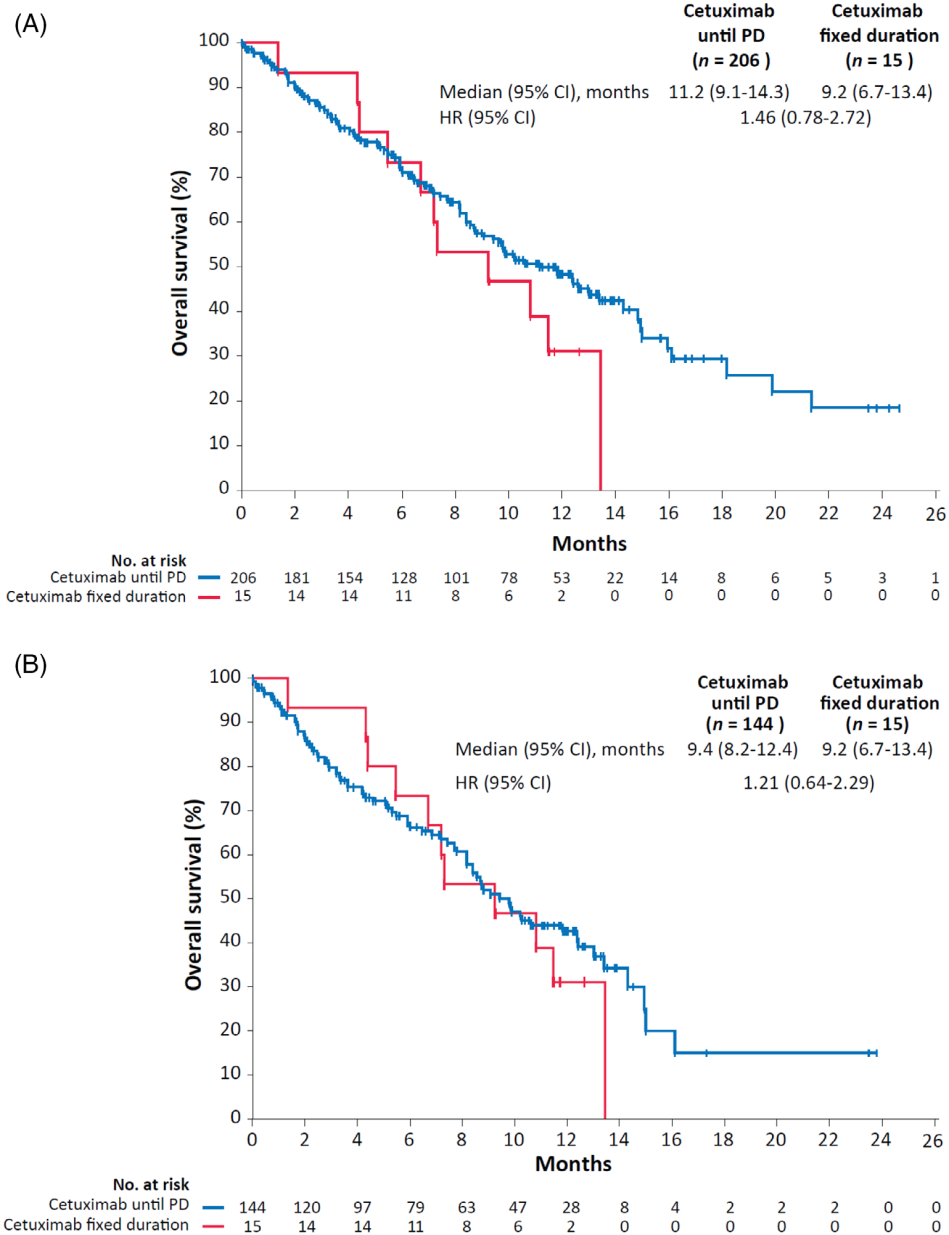
Kaplan–Meier estimates of overall survival for patients whose planned cetuximab treatment was until progressive disease (PD) or for a fixed duration in the full analysis set (A) and target analysis set (B). CI, confidence interval; HR, hazard ratio.

Median PFS was 6.5 months overall in the FAS (median PFS stratified by cetuximab duration was 6.8 months [cetuximab until PD] and 5.0 months [cetuximab fixed duration]; Figure 2A) and 5.2 months overall in the TAS (Figure 2B). Median OS was 10.8 months overall in the FAS (median OS stratified by cetuximab duration was 11.2 months [cetuximab until PD] and 9.2 months [cetuximab fixed duration]; Figure 3A) and 9.4 months overall in the TAS (Figure 3B).

No significant difference in PFS or OS was observed between the different countries. However, a significantly lower ORR was observed in patients from France (24.4%) and Italy (17.9%) compared with the other countries combined (46.7%; *p* values of .0485 and .0032, respectively). Furthermore, previous cetuximab therapy, chemotherapy regimen, or disease status did not impact efficacy results. In the multivariable Cox proportional hazard analysis for PFS, total number of sites with distant metastases (2 or ≥3 vs. 0–1), a higher baseline ECOG PS score (≥2 vs. 0–1) and the presence of nervous system disease were associated with a shorter duration of PFS (Supplementary Table 4). Similarly for OS, a higher baseline ECOG PS score (≥2 vs. 0–1) and nervous system disease were associated with a higher risk of death and shorter duration of OS. Conversely, greater body surface area was associated with a longer duration of OS (Hazard ratio 0.25 [95% CI 0.09, 0.75]; *p =* .0129) (Supplementary Table 5).

Exploratory analyses on the reasons for a treatment decision to be taken are shown in Supplementary Table 6. The mean ± standard deviation (STD) of the total scores for expected efficacy, expected quality of life and expected side effects (7.6 ± 2.03, 7.3 ± 2.08 and 6.2 ± 2.31, respectively) were higher than the mean ± STD for other scales (e.g., cost restrictions, 3.7 ± 3.29 or patients' preference, 4.5 ± 3.00). Multivariable logistic regression analysis of the association between planned treatment duration and the independent baseline variables of patient preference and cost restrictions is shown in Supplementary Table 7. Patient preference was found to be associated with a higher likelihood of treatment until PD than treatment for a fixed duration (Odds Ratio [OR] 2.72 [95% CI 1.52, 4.89]), whereas cost restrictions were associated with a higher likelihood of a fixed duration of treatment versus treatment until PD (OR 0.67 [95% CI 0.48, 0.93]).

### 3.4 Safety

In the safety analysis set (FAS; *N* = 221), AEs (within the specified AE categories that were collected in the study, including PD reported as an AE) occurred in 170 patients (76.9%) and were related to cetuximab in 104 patients (47.1%). The most frequent AE of any grade related to cetuximab was rash in 41 patients (18.6%), followed by asthenia in 15 patients (6.8%), skin reactions in 14 patients (6.3%), and dermatitis acneiform in 12 patients (5.4%) (Table 4). SAEs occurred in 85 patients (38.5%) and were considered related to cetuximab in 14 patients (6.3%). AEs that led to discontinuation of cetuximab occurred in 50 patients (22.6%). Sixty‐five patients (29.4%) had an AE that led to cetuximab dose delay, and 10 (4.5%) had an AE that led to cetuximab dose reduction. AEs leading to death occurred in 50 patients (22.6%).

**TABLE 4:** AEs of any grade related to cetuximab in ≥2% of patients in the FAS.

AE	*N* = 221
Patients with any AE related to cetuximab^a^	155 (70.1)
Rash	41 (18.6)
Asthenia	15 (6.8)
Skin toxicity	14 (6.3)
Dermatitis acneiform	12 (5.4)
Anemia	10 (4.5)
Mucosal inflammation	10 (4.5)
Skin fissures	9 (4.1)
Stomatitis	9 (4.1)
Nausea	8 (3.6)
Folliculitis	7 (3.2)
Diarrhea	6 (2.7)
Fatigue	6 (2.7)
Thrombocytopenia	5 (2.3)

Abbreviations: AE, adverse event; FAS, full analysis set.

^a^ Only serious AEs, non‐serious AEs related to study treatment, and AEs that caused treatment delay or permanent discontinuation were reported until the 30‐day posttreatment safety follow‐up assessment. Only these AEs that were related to cetuximab and occurred in ≥2% of patients in the FAS are summarized in this table.

Excluding PD, AEs occurred in 155 patients (70.1%) and were related to cetuximab in 102 patients (46.2%). Grade ≥3 AEs occurred in 88 patients (39.8%), and 24 patients (10.9%) had an AE leading to death. Fatal anaphylactic shock attributable to cetuximab occurred in one patient (0.5%) on the first day of treatment; all other AEs leading to death were not considered to be related to cetuximab.

## 4 DISCUSSION

The ENCORE study suggests that cetuximab plus PBT is an effective and widely used treatment in real‐world clinical settings across several countries according to current guidelines and continues to be a recommended 1L treatment for patients with R/M SCCHN. Of note, 45.2% of planned treatments did not include 5‐FU, and 3.2% included a taxane. Additionally, the median OS and PFS times in this study with 1L cetuximab plus platinum with/without 5‐FU were comparable to results with the EXTREME regimen (cetuximab plus platinum plus 5‐FU) in the randomized phase III EXTREME trial.^6^

The baseline characteristics of patients in the ENCORE study and the EXTREME trial were similar, although the median age was lower in the EXTREME trial (56 years) compared with the ENCORE study (64 years).^6^ Also, most patients in the ENCORE study had an ECOG PS of 0 or 1, whereas ECOG PS was not specified in the EXTREME trial; instead, patients were eligible if they had a Karnofsky performance status of ≥70.^6^ No new safety signals were observed with cetuximab plus platinum with/without 5‐FU compared with the EXTREME regimen, and cetuximab specifically was well tolerated, as shown by the low numbers of SAEs considered to be related to cetuximab and cetuximab dose delays/reductions/discontinuations, consistent with the known safety profile of cetuximab.

An important finding of the ENCORE study was that the planned cetuximab treatment duration for most patients with R/M SCCHN treated with cetuximab plus PBT was until PD (206 of 221 [93.2%]). In contrast, a very small proportion of patients (15 of 221 [6.8%]) were treated with a fixed duration of cetuximab. This indicates that treating clinicians would rather rely on treatment until PD, which was shown to be efficacious in the pivotal EXTREME trial.^6^ Interestingly, the exploratory analyses (Supplementary Tables 6 and 7) suggested that although the potential reasons for treatment decisions were usually based on expected efficacy, quality of life and side effects, patient preference was associated with a higher likelihood to treat until PD and cost restriction was associated with a higher likelihood to treat for a fixed period. The results should, however, be interpreted with caution, due to the low number of subjects planned to be treated for a fixed treatment period. Additionally, 59.7% of patients received cetuximab maintenance, suggesting that maintenance therapy with cetuximab after stopping PBT is widely used. The ENCORE study also showed that cetuximab plus PBT was feasible in an unselected patient population and was adaptable to suit patient needs (e.g., almost half of the patients did not receive 5‐FU).

The EXTREME regimen was recently evaluated in patients with R/M SCCHN who were ≥70 years old in the ELAN (ELderly head And Neck cancer) trials.^14^ ELAN was a large prospective clinical program designed to improve the management of elderly patients with SCCHN by using an adapted geriatric evaluation that is feasible for use in daily practice. In the ELAN FIT trial, 78 patients were treated with the EXTREME regimen with efficacy assessed by the ORR at 12 weeks and safety assessed by lack of grade ≥4 toxicity and no loss of independence. The 12‐week ORR was 38% in fit elderly patients with SCCHN. Other efficacy outcomes included a median OS of 14.7 months (95% CI, 11.0–18.2 months) and a median PFS of 7.1 months (95% CI, 5.5–8.2 months); nearly a quarter (24%) of the patients experienced a grade ≥4 AE. Benefit was observed in elderly patients determined as fit by using the ELAN geriatric evaluation criteria.^14^ The results of ENCORE compare favorably with these outcomes; thus, these data establish the utility of cetuximab plus PBT across a variety of patient populations.

There were limitations associated with the ENCORE study, which are typical of the observational study design. Some patients began cetuximab treatment before the date of enrollment, potentially leading to selection bias and study outcomes bias. Their outcomes may have a positive bias, particularly for time‐to‐event endpoints, because patients who were already on treatment without progression upon enrollment were more likely to be responders and have longer survival times than patients starting treatment at enrollment. The TAS was introduced for those patients in the FAS who had only started cetuximab after their enrollment. The difference between the results in the FAS and TAS allows an assessment of the effect of the selection bias. Efficacy outcomes from the TAS may therefore be considered more reliable than those of the FAS. Furthermore, conducting the safety analysis on the TAS may be more representative of real‐world clinical practice as any AEs occurring before study enrollment may not be captured in the FAS, potentially underestimating treatment toxicity. Another limitation was that patients were enrolled from only 6 countries (Algeria, France, Italy, Portugal, Russia, and South Africa); therefore, a true global perspective of real‐world practices for 1L R/M SCCHN treatment may not have been gained. The low number of patients whose treatment was planned for a fixed duration does not allow a valid conclusion regarding whether the planned treatment duration (until PD or for a fixed duration) had an impact on the treatment outcome; however, this analysis does show that treatment until PD is a well‐accepted regimen, consistent with results from the EXTREME study.^6^ In the real‐world clinical setting, HPV status was assessed in 28.6% of patients with oropharyngeal cancer, which may have been because HPV testing was not considered routine for R/M SCCHN in all countries included in this study during the enrollment period. However, it is interesting to note that analysis of the impact of HPV and p16 status in the EXTREME trial showed that although HPV and tumor p16 positivity have prognostic value in R/M SCCHN, the survival benefits of cetuximab plus chemotherapy compared with chemotherapy alone were independent of p16 or HPV status.^15^ Moreover, only 5% of the patients tested were HPV positive (416 tested out of 442 total patients in the EXTREME trial), which is not unexpected given the better prognosis for patients with HPV positive and locally advanced SCCHN.^15^ Since the ENCORE trial was conducted in a similar European patient population as the EXTREME trial, it is likely that the prevalence of HPV positivity is similarly not very high in this R/M SCCHN patient population, and that HPV status may not have an impact on outcomes for the cetuximab + PBT regimens. Additionally, as only SAEs, non‐serious AEs related to study treatment, and AEs leading to a delay or the permanent discontinuation of study treatment were recorded by each treating physician, a full safety profile of cetuximab plus PBT was not reported in this study. Furthermore, 45.2% of patients had planned treatments that did not include 5‐FU and were therefore not receiving the full EXTREME regimen. It is possible that 5‐FU was not included due to comorbidities or the frailty/vulnerability of elderly patients with SCCHN^16^ (median age in this study was 64 years); however, the reasons why the planned treatments for some patients did not include 5‐FU were not recorded.

In summary, ENCORE provides insight into real‐world clinical practices for 1L R/M SCCHN treatment and further contributes to the growing body of evidence suggesting that cetuximab plus PBT is a safe and effective treatment across a broad patient population. In an international real‐world setting, median OS and PFS times with 1L cetuximab plus platinum with/without 5‐FU were comparable to the results observed in the randomized pivotal phase III EXTREME trial.^6^ On the basis of these outcomes, cetuximab plus PBT should continue to be a treatment option for patients with 1L R/M SCCHN.

## AUTHOR CONTRIBUTIONS

**Christophe Le Tourneau:** Conceptualization (equal); data curation (equal); formal analysis (equal); investigation (equal); resources (equal); writing – original draft (equal). **Massimo Ghiani:** Conceptualization (equal); data curation (equal); formal analysis (equal); investigation (equal); resources (equal); writing – original draft (equal). **Maria Chiara Cau:** Conceptualization (equal); data curation (equal); formal analysis (equal); investigation (equal); resources (equal); writing – original draft (equal). **Roberta Depenni:** Conceptualization (equal); data curation (equal); formal analysis (equal); investigation (equal); resources (equal); writing – original draft (equal). **Graziana Ronzino:** Conceptualization (equal); data curation (equal); formal analysis (equal); investigation (equal); resources (equal); writing – original draft (equal). **Pierluigi Bonomo:** Conceptualization (equal); data curation (equal); formal analysis (equal); investigation (equal); resources (equal); writing – original draft (equal). **Vincenzo Montesarchio:** Conceptualization (equal); data curation (equal); formal analysis (equal); investigation (equal); resources (equal); writing – original draft (equal). **Luigi Leo:** Conceptualization (equal); data curation (equal); formal analysis (equal); investigation (equal); resources (equal); writing – original draft (equal). **Jeltje Schulten:** Conceptualization (equal); formal analysis (equal); resources (equal); writing – original draft (equal). **Satu Salmio:** Conceptualization (equal); formal analysis (equal); resources (equal); writing – original draft (equal). **Diethelm Messinger:** Conceptualization (equal); formal analysis (equal); writing – original draft (equal). **Andrea Sbrana:** Conceptualization (equal); data curation (equal); formal analysis (equal); investigation (equal); resources (equal); writing – original draft (equal). **Edith Borcoman:** Conceptualization (equal); data curation (equal); formal analysis (equal); investigation (equal); resources (equal); writing – original draft (equal). **Maria Grazia Ghi:** Conceptualization (equal); data curation (equal); formal analysis (equal); investigation (equal); resources (equal); writing – original draft (equal).

## CONFLICT OF INTEREST STATEMENT

CLT reports receiving honoraria and travel expenses and providing consulting or advisory services for AstraZeneca, Bristol Myers Squibb, Celgene, EMD Serono, GlaxoSmithKline, Merck & Co., Nanobiotix, Rakuten Medical, Roche, and Seattle Genetics. PB reports providing consulting or advisory services for Angelini Pharma and EMD Serono. VM reports receiving honoraria from Bristol‐Myers Squibb and Italfarmaco. JS and SS are employees of the healthcare business of Merck KGaA, Darmstadt, Germany. DM was an employee of Prometris GmbH. All other authors report no conflicts of interest.

## ETHICS STATEMENT

All patients provided written informed consent prior to enrollment, and the protocol was approved by the local regulatory and ethics committee at each participating center. The study was conducted in accordance with the Declaration of Helsinki and the International Council on Harmonization Guidelines on Good Clinical Practice.

## Supporting information


**Supplementary Table 1:** Planned duration of chemotherapy in the FAS.
**Supplementary Table 2:** Cetuximab treatment exposure in the FAS.
**Supplementary Table 3:** Cetuximab cumulative dose and relative dose intensity in the FAS†, ‡.
**Supplementary Table 4:** Multivariable Cox proportional hazards model to identify prognostic factors for PFS in the TAS.
**Supplementary Table 5:** Multivariable Cox proportional hazards model to identify prognostic factors for OS in the TAS.
**Supplementary Table 6:** Reasons (ratings) for planned treatment (FAS).
**Supplementary Table 7:** Association between baseline and disease characteristics and planned cetuximab treatment duration – multiple logistic regression analysis (TAS).

## Data Availability

Any requests for data by qualified scientific and medical researchers for legitimate research purposes will be subject to the Data Sharing Policy of the healthcare business of Merck KGaA, Darmstadt, Germany. All requests should be submitted in writing to the data sharing portal of the healthcare business of Merck KGaA, Darmstadt, Germany (https://www.merckgroup.com/en/research/our-approach-to-research-and-development/healthcare/clinical-trials/commitment-responsible-data-sharing.html). When the healthcare business of Merck KGaA, Darmstadt, Germany has a co‐research, co‐development, or co‐marketing or co‐promotion agreement, or when the product has been out‐licensed, the responsibility for disclosure might be dependent on the agreement between parties. Under these circumstances, the healthcare business of Merck KGaA, Darmstadt, Germany will endeavor to gain agreement to share data in response to requests.
