# Supplementary material for: First‐line cetuximab + platinum‐based therapy for recurrent/metastatic head and neck squamous cell carcinoma: A real‐world observational study—ENCORE
Source: Cancer Rep (Hoboken). 2023 Apr 17;6(5):e1804. doi: 10.1002/cnr2.1804 (PMC10172179; doi:10.1002/cnr2.1804)
Supplement: Supplementary file 1 — Supplementary Table 1: Planned duration of chemotherapy in the FAS. Supplementary Table 2: Cetuximab treatment exposure in the FAS. Supplementary Table 3: Cetuximab cumulative dose and relative dose intensity in the FAS†, ‡. Supplementary Table 4: Multivariable Cox proportional hazards model to identify prognostic factors for PFS in the TAS. Supplementary Table 5: Multivariable Cox proportional hazards model to identify prognostic factors for OS in the TAS. Supplementary Table 6: Reasons (ratings) for planned treatment (FAS). Supplementary Table 7: Association between baseline and disease characteristics and planned cetuximab treatment duration – multiple logistic regression analysis (TAS). [file CNR2-6-e1804-s001.docx]

**Supplementary Material**

**Supplementary Table 1.** Planned duration of chemotherapy in the FAS

|  | **Patients, *n* (%)** | | |
| --- | --- | --- | --- |
| **Planned chemotherapy duration (if not until PD), weeks** | **Cetuximab until PD (*n* = 206)** | **Cetuximab fixed duration (*n* = 15)** | **Total (*N* = 221)** |
| 1 | 0 | 2 (13.3) | 2 (0.9) |
| 3 | 2 (1.0) | 1 (6.7) | 3 (1.4) |
| 4 | 0 | 1 (6.7) | 1 (0.5) |
| 6 | 18 (8.7) | 2 (13.3) | 20 (9.0) |
| 9 | 2 (1.0) | 0 | 2 (0.9) |
| 12 | 10 (4.9) | 5 (33.3) | 15 (6.8) |
| 16 | 2 (1.0) | 0 | 2 (0.9) |
| 18 | 59 (28.6) | 1 (6.7) | 60 (27.1) |
| 24 | 1 (0.5) | 3 (20.0) | 4 (1.8) |
| 30 | 1 (0.5) | 0 | 1 (0.5) |

Abbreviations: FAS, full analysis set; PD, progressive disease.

**Supplementary Table 2.** Cetuximab treatment exposure in the FAS

| **Outcome, *n* (%)** | **Cetuximab until PD**  **(*n* = 206)** | **Cetuximab fixed duration**  **(*n* = 15)** | **Total (*N* = 221)** |
| --- | --- | --- | --- |
| **Cetuximab dose reduced** |  |  |  |
| Yes† | 19 (9.2) | 2 (13.3) | 21 (9.5) |
| No | 187 (90.8) | 13 (86.7) | 200 (90.5) |
| Missing | 0 | 0 | 0 |
| **Cetuximab dose permanently discontinued** |  |  |  |
| Yes | 160 (77.7) | 14 (93.3) | 174 (78.7) |
| No | 28 (13.6) | 1 (6.7) | 29 (13.1) |
| Missing | 18 (8.7) | 0 | 18 (8.1) |
| **Reason for discontinuation of cetuximab** |  |  |  |
| PD | 70 (34.0) | 2 (13.3) | 72 (32.6) |
| Unrelated AE | 6 (2.9) | 0 | 6 (2.7) |
| Related AE | 3 (1.5) | 2 (13.3) | 5 (2.3) |
| SAE | 12 (5.8) | 0 | 12 (5.4) |
| According to planned fixed duration | 8 (3.9) | 5 (33.3) | 13 (5.9) |
| Patient refused further treatment | 6 (2.9) | 0 | 6 (2.7) |
| Patient nonattendance | 8 (3.9) | 0 | 8 (3.6) |
| Lost to follow-up | 7 (3.4) | 0 | 7 (3.2) |
| Investigator decision | 21 (10.2) | 4 (26.7) | 25 (11.3) |
| Sufficient response | 8 (3.9) | 0 | 8 (3.6) |
| Death | 10 (4.9) | 1 (6.7) | 11 (5.0) |
| Missing | 1 (0.5) | 0 | 1 (0.5) |

Abbreviations: AE, adverse event; FAS, full analysis set; PD, progressive disease; SAE, serious adverse event.

† If cetuximab revised dose was less than the dose administered at baseline.

**Supplementary Table 3.** Cetuximab cumulative dose and relative dose intensity in the FAS†,‡

| Variable | cetuximab until PD  n=206 | cetuximab not until PD  n=15 | Total  N=221 |
| --- | --- | --- | --- |
| Total number of cetuximab administrations per subject |  |  |  |
| n (%) | 206 (100.0) | 15 (100.0) | 221 (100.0) |
| Mean ± STD | 14.2 ± 13.19 | 11.8 ± 7.98 | 14.1 ± 12.91 |
| Median | 11.0 | 12.0 | 11.0 |
| Cumulative dose of cetuximab (mg/m²) |  |  |  |
| n (%) | 206 (100.0) | 15 (100.0) | 221 (100.0) |
| Mean ± STD | 4108.5 ± 3736.98 | 2995.3 ± 2011.38 | 4032.9 ± 3653.64 |
| Median | 2900.0 | 3150.0 | 2900.0 |
| Relative Dose Intensity for cetuximab (%) |  |  |  |
| n (%) | 186 (90.3) | 14 (93.3) | 200 (90.5) |
| Mean ± STD | 71.3 ± 31.05 | 80.5 ± 23.39 | 72.0 ± 30.62 |
| Median | 77.0 | 88.5 | 77.5 |

Abbreviations: PD, progressive disease; STD, standard deviation

† Denominator for % calculation based on total number of subjects in each respective group from Full Analysis Set.

‡ Summary of cumulative dose and dose intensity for the various chemotherapies used was not possible because the exact dose was not documented in the patient case report form if dose modifications had occurred.

**Supplementary Table 4.** Multivariable Cox proportional hazards model to identify prognostic factors for PFS in the TAS

| Independent variable | Category | Progression of Disease or Death | | N=159 | | | | |
| --- | --- | --- | --- | --- | --- | --- | --- | --- |
|  |  | Yes | No | Regression  Coefficient | Standard  Error | *P*-value | Hazard Ratio  (95% CI) | Effect *P* -value† |
| Total number of sites with distant metastasis | 0-1 | 104 | 30 | - | - | - | 1.00 | - |
|  | 2 | 16 | 2 | 0.6889 | 0.2903 | 0.0176 | 1.99 (1.13, 3.52) | - |
|  | ≥ 3 | 5 | 0 | 0.5909 | 0.4750 | 0.2134 | 1.81 (0.71, 4.58) | 0.0414 |
| Baseline ECOG PS score | 0-1 | 105 | 31 | - | - | - | 1.00 | - |
|  | ≥ 2 | 20 | 1 | 0.7982 | 0.2659 | 0.0027 | 2.22 (1.32, 3.74) | 0.0027 |
| Nervous System Disease (incl. peripheral neuropathy) | Yes | 10 | 0 | - | - | - | 1.00 | - |
|  | No | 115 | 32 | -1.0047 | 0.3398 | 0.0031 | 0.37 (0.19, 0.71) | 0.0031 |

Abbreviations: CI, confidence interval; ECOG PS, Eastern Cooperative Oncology Group Performance Status

† Wald chi-square test for the null hypothesis that all regression coefficients of an independent variable were zero.

**Supplementary Table 5.** Multivariable Cox proportional hazards model to identify prognostic factors for OS in the TAS

| Independent variable | Category | Death from any cause | | N=159 | | | | |
| --- | --- | --- | --- | --- | --- | --- | --- | --- |
|  |  | Yes | No | Regression  Coefficient | Standard  Error | *P* -value | Hazard Ratio  (95% CI) | Effect *P* -value† |
| BSA (m^2^) | - | 86 | 69 | -1.3781 | 0.5541 | 0.0129 | 0.25 (0.09, 0.75) | 0.0129 |
| Baseline ECOG PS score | 0-1 | 68 | 66 | - | - | - | 1.00 | - |
|  | ≥ 2 | 18 | 3 | 1.1613 | 0.2745 | <0.0001 | 3.19 (1.87, 5.47) | <0.0001 |
| Nervous System Disease (incl. peripheral neuropathy) | Yes | 7 | 3 | - | - | - | 1.00 | - |
|  | No | 79 | 66 | -1.0062 | 0.4049 | 0.0129 | 0.37 (0.17, 0.81) | 0.0129 |

Abbreviations: BSA, Body Surface Area; CI, confidence interval; ECOG PS, Eastern Cooperative Oncology Group Performance Status

† Wald chi-square test for the null hypothesis that all regression coefficients of an independent variable were zero.

Supplementary Table 6: Reasons (ratings) for planned treatment (FAS)

|  | **Cetuximab until PD**  **N=206 (100%)** | **Cetuximab not until PD**  **N=15 (100%)** | **Total**  **N=221 (100%)** |
| --- | --- | --- | --- |
| Efficacy expected (0 to 10) |  |  |  |
| n (%) | 206 (100.0) | 15 (100.0) | 221 (100.0) |
| Mean ± STD | 7.6 ± 1.97 | 7.2 ± 2.76 | 7.6 ± 2.03 |
| Median | 8.0 | 8.0 | 8.0 |
| Quality of life expected (0 to 10) |  |  |  |
| n (%) | 206 (100.0) | 15 (100.0) | 221 (100.0) |
| Mean ± STD | 7.3 ± 2.01 | 6.2 ± 2.65 | 7.3 ± 2.08 |
| Median | 7.0 | 8.0 | 7.0 |
| Side effects expected (0 to 10) |  |  |  |
| n (%) | 206 (100.0) | 15 (100.0) | 221 (100.0) |
| Mean ± STD | 6.4 ± 2.15 | 3.9 ± 3.26 | 6.2 ± 2.31 |
| Median | 7.0 | 5.0 | 7.0 |
| Length of treatment expected (0 to 10) |  |  |  |
| n (%) | 206 (100.0) | 15 (100.0) | 221 (100.0) |
| Mean ± STD | 5.5 ± 2.89 | 3.1 ± 2.70 | 5.3 ± 2.93 |
| Median | 6.0 | 3.0 | 6.0 |
| Mode of administration (0 to 10) |  |  |  |
| n (%) | 206 (100.0) | 15 (100.0) | 221 (100.0) |
| Mean ± STD | 5.0 ± 2.27 | 4.6 ± 4.17 | 5.0 ± 2.43 |
| Median | 5.0 | 5.0 | 5.0 |
| Subject preference (0 to 10) |  |  |  |
| n (%) | 206 (100.0) | 15 (100.0) | 221 (100.0) |
| Mean ± STD | 4.7 ± 2.93 | 1.3 ± 1.75 | 4.5 ± 3.00 |
| Median | 5.0 | 0.0 | 5.0 |
| Cost restrictions (0 to 10) |  |  |  |
| n (%) | 206 (100.0) | 15 (100.0) | 221 (100.0) |
| Mean ± STD | 3.8 ± 3.29 | 2.5 ± 3.11 | 3.7 ± 3.29 |
| Median | 5.0 | 1.0 | 4.0 |

Abbreviations: FAS, full analysis set; PD, Disease Progression; STD, Standard Deviation.

Rating was done between '0' to '10'; '0' suggests 'Least important' and '10' suggests 'Most important'. Denominator for % calculation based on total number of subjects in each respective group.

**Supplementary Table 7:** Association between baseline and disease characteristics and planned cetuximab treatment duration – multiple logistic regression analysis (TAS)

| Independent variable | Category | Cetuximab treatment planned until PD | | N=159 | | | | |  |
| --- | --- | --- | --- | --- | --- | --- | --- | --- | --- |
|  |  | Yes | No | Regression  Coefficient | | Standard  Error | p-value | Odds Ratio  (95% CI) | Effect  p-value^#^ |
| **Cost restrictions** (0 to 10)* | Per 1 point increment | 142 | 15 | -0.4022 | 0.1669 | | 0.0160 | 0.67 (0.48, 0.93) | 0.0160 |
| **Subject preference** (0 to 10)* | Per 1 point increment | 142 | 15 | 1.0019 | 0.2988 | | 0.0008 | 2.72 (1.52, 4.89) | 0.0008 |
| **Treatment goal** | Symptomatic treatment | 4 | 5 |  |  | |  | 1.00 |  |
|  | Palliative | 49 | 3 | 2.4602 | 1.0061 | | 0.0145 | 11.71 (1.63, 84.11) |  |
|  | Potentially Curative | 89 | 7 | 2.6326 | 1.1841 | | 0.0262 | 13.91 (1.37, 141.64) | 0.0410 |

Abbreviations: PD, Disease Progression; TAS, Target analysis set.

*Rating was done between '0' to '10'; '0' suggests 'Least important' and '10' suggests 'Most important'

^#^Wald chi-square test for the null hypothesis that all regression coefficients of an independent variable were zero.
